# Gestational weight gain and body composition of full-term newborns and infants: a cohort study

**DOI:** 10.1186/s12884-020-03145-x

**Published:** 2020-08-20

**Authors:** Sylvia R. Nehab, Letícia D. Villela, Fernanda V. M. Soares, Andrea D. Abranches, Daniele M. R. Araújo, Leila M. L. da Silva, Yasmin N. V. Amaral, Saint Clair G. Junior, Maria Dalva B. B. Meio, Maria Elisabeth Moreira

**Affiliations:** Instituto Fernandes Figueira – Fiocruz, Avenida Rui Barbosa 716- Flamengo, Rio de Janeiro, RJ Brazil

**Keywords:** Adiposity, Body composition, Gestational weight gain, Pregnancy

## Abstract

**Background:**

The association between gestational weight gain and neonatal body composition has been inconsistent, exposing the need for further research. The aim of this study was to evaluate whether gestational weight gain influences the body composition of full-term newborns and infants up to 4 months old.

**Methods:**

A cohort study was performed with 124 participants divided into categories of gestational weight gain according to the 2009 Institute of Medicine guidelines. The anthropometric and body composition data of newborns and infants acquired using air displacement plethysmography (PeaPod®) were collected at 96 h, 1 month, 2 months and 4 months of life. In the statistical analysis, the chi-square test was used to analyze categorical variables, and ANOVA was used to analyze numerical variables. Univariate analysis was performed, and the absolute and relative frequencies of the categorical variables, as well as mean and standard deviation of the numerical variables, were obtained. Bivariate analysis was performed for the categories of gestational weight gain and gestational and neonatal characteristics. When adjustments to gestational hypertension, gestational diabetes, and pregestational body mass index (BMI) were analyzed by linear regression, gestational weight gain remained a significant variable for newborn percent fat mass. For all analyses, a significance level of 5% was adopted.

**Results:**

Gestational weight gain was adequate in 33.8% of the participants, excessive in 41.1% and insufficient in 25%. Women with excessive weight gain had higher pregestational BMIs and a higher incidence of gestational hypertension. Their newborns had a higher body mass, body fat mass in grams and percent fat mass than the infants born to mothers with adequate or insufficient gestational weight gain. No significant differences were observed in body composition at 1, 2 and 4 months of life during infant follow-up.

**Conclusion:**

Excessive gestational weight gain may alter the body composition of newborns at birth. Further studies are required to better evaluate infant follow-up.

**Trial registration:**

Clinical Trial Registry: NCT00875251 on April 3, 2009.

## Background

The prevalence of obesity has been increasing at an epidemic rate over the last 30 years [1]. WHO data indicate that 39% of adults older than 18 years of age were overweight and 13% were obese in 2016. Among children under 5 years old, 41 million were overweight or obese. In Brazil, more than 30% of children aged 5–10 years of age are overweight, and approximately 14% are obese [2].

The course of obesity can begin very early, especially in utero and in the first 2 years of life [3, 4]. An adverse intrauterine environment may increase the risk for obesity and, consequently, their related pathologies, such as cardiovascular and metabolic diseases, including dyslipidemia, hypertension, and insulin resistance [5, 6].

A meta-analysis and systematic review revealed an increased risk of macrosomia for newborns of mothers with excessive gestational weight gain [7]. However, studies that evaluated the association between gestational weight gain and infant body composition revealed inconsistent results, exposing the need for further research [8, 9].

Body composition at birth may predict long-term effects, suggesting that the body fat mass (FM) and fat-free mass (FFM) in fetal and neonatal life influence long-term cardiometabolic risk [10–12]. Thus, this study aimed to evaluate whether gestational weight gain affects the body composition of full-term newborns.

## Methods

This prospective cohort study was carried out at the Fernandes Figueira National Institute of Women, Children and Adolescent Health, Oswaldo Cruz Foundation, Rio de Janeiro (RJ), Brazil. The study was approved by the IRB CEPIFF from Instituto Fernandes Figueira/Fiocruz Human Research Ethics Committee (CAAE 50773615.5.1001.526). Parent consent forms were obtained before data was collected from the newborn and infants. The Clinicaltrials.gov registration number is NCT00875251.

The study included mothers and their newborns born during gestational week 37 or later and hospitalized in the well-baby nursery room of the Institute’s hospital from March 2016 to August 2017. Patients with congenital malformations, genetic syndromes, exposure to TORCH congenital infections, human immunodeficiency virus and Zika virus, multiple gestations, and newborns with blood incompatibility who required phototherapy were excluded from the study.

Gestational age at birth was determined based on the first-trimester ultrasonography or the date of the last menstrual period [13].

The exposure variable was gestational weight gain, calculated as the difference between the pregestational weight and weight at the last prenatal visit before birth (38 ± 2 weeks). Both weights were obtained from prenatal records. The pregestational weight is the last known weight before knowledge of the pregnancy and was provided by the woman at her first prenatal appointment.

The body mass index (BMI) was obtained by dividing the mother’s pre-pregnancy weight by height squared (kg/m^2^) [10]. This criterion establishes the following: for low-weight women (BMI < 18.5 kg/m^2^), the appropriate gestational weight gain is from 12.5 to 18 kg; for eutrophic women (BMI: 18.5–24.9 kg/m^2^), 11.5 to 15.9 kg; for overweight women (BMI: 25.0–29.9 kg/m^2^), 7 to 11.5 kg; and for obese women (BMI ≥ 30.0 kg/m^2^), 5 to 9 kg. Gestational weight gain was then classified as insufficient, appropriate or excessive according to the pregestational BMI based on the 2009 Institute of Medicine (IOM) criteria [10].

Newborn and infant growth and body composition were evaluated in the first 96 h of life and at 1, 2 and 4 months of life. Trained researchers evaluated each infant’s weight, height, head circumference (grams and Z score) and BMI, as well as FM and FFM (in grams and %).

Air displacement plethysmography (PeaPod Infant Body Composition System Life Measurement, Inc., Concord Canada, CA) was used to estimate the body composition by densitometry. Body density was used in a two-compartment model to calculate the percentage of fat mass and fat-free mass. By definition, the density of the whole body is the body mass divided by body volume. The PeaPod® is a noninvasive technique that is not only accurate and easily used by operators but also comfortable and adequate for serial evaluations of infants between birth and 6 months of age or for infants weighing up to 8 kg [14, 15]. The weight in grams was obtained using the PeaPod® high precision scale. Body length (in centimeters) was measured on a specific anthropometric scale. The head circumference (in centimeters) was measured in millimeters using an inextensible measuring tape that was well-adjusted on the supraorbital region, in front of the head and over the occipital prominence, and on the back of the head. INTERGROWTH recommendations were used for the anthropometric measurements [16].

The Z-score was calculated for the weight/age, height/age, and head circumference/age indexes based on growth curves proposed by the WHO in 2006 using WHO Anthro software (version 3.2.2 January 2011).

Data on the newborns and infants, such as gestational age, birth weight, sex, Apgar score and type of feeding, and maternal data, such as demographic data, occupational data, educational level, socioeconomic status, presence of chronic diseases during gestation, type of delivery, parity and smoking status, were collected from the information recorded in the medical records and prenatal card and through interviews with the puerperal women.

Gestational hypertension was defined as a systolic blood pressure ≥ 140 mmHg or diastolic blood pressure ≥ 90 mmHg at two different times during gestation [17, 18]. Gestational diabetes mellitus was characterized using the following cutoff points: fasting plasma glucose ≥92 mg/dl; one-hour plasma glucose, after the oral glucose tolerance test (OGTT) ≥ 180 mg/dl; or two-hour plasma glucose, after OGTT ≥153 mg/dl, at any time during gestation [19, 20].

A sample size of 124 participants was calculated considering the results observed by *Hull* et al.^*21*^ for the neonatal % fat mass (mean 11.2 ± 5,3% fat mass in the appropriate gestational weight gain group and 12,7 ± 4,6% fat mass in the excessive gestational weight gain group); the difference between the groups was 2,5% fat mass. An 80% power and 95% confidence interval were considered.

Data storage was performed using EpiData software version 3.1. The SPSS 22 program was used for data treatment and all statistical analyses.

Descriptive analysis was performed with absolute and relative frequencies for the categorical variables and measurements of central tendency and dispersion for the numerical variables. Bivariate analysis was carried out using the categories of gestational weight gain with maternal and neonatal characteristics. To evaluate the statistically significant associations, the chi-square test was used for categorical variables, and analysis of variance (ANOVA) was used for numerical variables. A linear regression model was performed to evaluate the relationship between maternal factors (gestational weight gain, gestational diabetes, gestational hypertension, and pregestational BMI) and neonatal FM and FFM. For all analyses, a significance level of 0.05 was used to identify statistically significant differences.

## Results

**Fig. 1:**
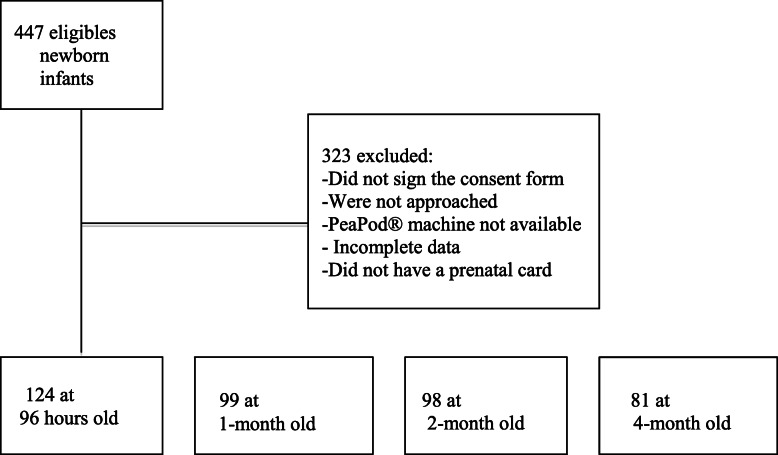
Flowchart of the study cohort

One hundred twenty-four mothers and their newborn infants were included in this study, and 81 pairs remained in the study after 4 months (Fig. 1).

Regarding pregestational nutritional status, 4% of the participants were considered low weight, 49% had an adequate weight, 33% were overweight, and 13.7% were obese.

The mothers were divided into three groups according to gestational weight gain following the 2009 IOM guidelines: appropriate (33.8%), excessive (41,1%) and insufficient (25%).

Maternal age, ethnicity, marital status, percentage working outside the home, smoking status, number of prenatal visits, proportion of cesarean deliveries, and incidence of gestational diabetes were similar among groups (Table 1). Socioeconomic status was considered in this study, and we observed no significant differences among groups in terms of income or education level.

**Table 1:** Maternal and gestational characteristics in different groups of gestational weight gain according to IOM criteria, (*n* = 124)

Mother characteristics	Gestational Weight Gain			*p*-value
	Insufficient^c^ (*n* = 31)	Appropriate^a^ (*n* = 42)	Excessive ^b^ (*n* = 51)	
	Mean (±SD) or n (%)	Mean (± SD) or n (%)	Mean (±SD) or n (%)	
Maternal age (years)	29.4 (± 7.98)	29.81 (± 6.71)	28.12 (± 7.20)	0.503
Number of prenatal visits	9.10 (± 2.83)	12.54 (± 14.45)	9.63 (± 2.14)	0.181
Pre-gestational BMI (kg/m^2^)	23.99 (± 5.23)	24.31 (± 4.06)	27.01 (± 5.07)	0.006
White	11 (35.5%)	17 (40.5%)	20 (40.0%)	0.624
Works outside the home	12 (38.7%)	20 (47.6%)	23 (45.1%)	0.743
Smoker	2 (6.5%)	0 (0%)	3 (5.9%)	0.261
Married	24 (80.0%)	39 (92.9%)	42 (82.4%)	0.481
C-section	14 (45.2%)	20 (47.6%)	27 (52.9%)	0.653
Gestational hypertension	6 (19.4%)	7 (16.7%)	25 (49.0%)	0.001
Gestational diabetes	8 (25.8%)	5 (11.9%)	7 (13.7%)	0.232
Pre-gestational nutritional status				0.121
Low weight	3 (9.7%)	1 (2.4%)	1 (2.0%)	
Eutrophic	18 (58.1%)	24 (57.1%)	19 (37.3%)	
Overweight	7 (22.6%)	13 (31.0%)	21 (41.2%)	
Obese	3 (9.7%)	4 (9.5%)	10 (19.6%)	

^a^ Gestational weight gain within 2009 IOM recommendations

^b^ Gestational weight gain more significant than the 2009 IOM recommendations

^c^ Weight gain less than the 2009 IOM recommendations

Recommendations from 2009 IOM. Pregnant women with low pre-gestational weight, the recommended weight gain is 12.5 to 18 kg; eutrophic, 11.5 to 16 kg; overweight, 7 to 11.5 kg; and obese from 5 to 9 kg

Women with excessive gestational weight gain had a higher pregestational BMI and a higher percentage of gestational hypertension than those in the other groups (*p* < 0.005) (Table 1).

Newborn infants of mothers with excessive gestational weight gain demonstrated an increased birth weight as well as FM and % FM within the first 96 h compared with those born to mothers with appropriate or insufficient gestational weight gain (Table 2). The Z-scores of weight and head circumference for age and BMI were also increased in the group of newborns with mothers who had excessive weight gain during pregnancy (Table 2). When adjustments to gestational hypertension, gestational diabetes, and pregestational BMI were analyzed by linear regression, gestational weight gain remained a significant variable for newborn % FM and FFM.

**Table 2:** Neonatal characteristics in different groups of gestational weight gain (*n* = 124)

Neonatal features	Gestational Weight Gain			*p*-value
	Insufficient^**c**^ (*n* = 31)	Appropriate ^**a**^ (*n* = 42)	Excessive^**b**^ (*n* = 51)	
	Mean (± SD) or n (%)	Mean (± SD) or n (%)	Mean (± SD) or n (%)	
Gender (male)	17 (54.8%)	20 (47.6%)	28 (54.9%)	0.746
Gestational Age (weeks)	39.74 (± 0.99)	38.64 (± 1.34)	38.90 (± 1.41)	0.625
Birth weight (g)	3134.84 (± 393.84)	3220.00 (± 448.33)	3421.57 (± 487.43)	0.014
HC (cm)	34.44 (± 1.20)	34.28 (± 0.99)	34.90 (± 1.11)	0.022
Height (cm)	49.17 (± 1.89)	49.22 (± 2.03)	50.01 (± 1.96)	0.083
Apgar 5 min	9	9	9	0.47
Z weight/age	−0.73 (± 0.83)	−0.66 (± 0.91)	−0.19 (± 0.99)	0.017
Z HC/age	−0.02 (± 1.02)	−0.08 (± 0.86)	0.37 (± 0.94)	0.040
Z length/age	−0.44 (± 0.98)	−0.37 (± 1.11)	0.01 (± 1.04)	0.092
BMI	12.23 (± 0.91)	12.28 (± 0.91)	12.88 (± 1.18)	0.005
% Fat-free mass	91.35 (± 4.25)	91.15 (± 3.96)^#^	89.13 (± 4.73)^##^	0.035
Fat-free mass (g)	2716.03 (± 317.44)	2717.07 (± 301.33)	2858.04 (± 364.61)	0.074
% Fat mass	8.64 (± 4.25)	8.85 (± 3.95)^#^	10.86 (± 4.73)^##^	0.035
Fat mass (g)	264.70 (± 140.90)	275.20^#^ (± 146.57)	359.82^##^ (± 181.53)	0.013

^#^Appropriate x excessive (*p* < 0.05) / ^##^Excessive x insufficient (*p* < 0.05)

*BMI* Body mass index

*HC* Head circumference

^a^ Gestational weight gain within 2009 IOM recommendations

^b^ Gestational weight gain greater than the 2009 IOM recommendations

^c^ Weight gain less than the 2009 IOM recommendations

There were no differences in infant growth, body composition or type of feeding at 1, 2 and 4 months of age (*p*-value > 0,05) (Table 3).

**Table 3:** Data on feeding, growth and body composition of infants with 1, 2 and 4 months of life, (mean and standard deviation or number and percentage)

Characteristics of infants	Gestational Weight Gain			
Month of evaluation	Insufficient	Appropriate	Excessive	
**1 month**	(*n* = 25)	(*n* = 38)	(*n* = 36)	*p*-value
Exclusive Breastfeeding	21 (84.0%)	29 (76.3%)	29 (80.6%	0.751
Breastfeeding + formula	4 (16.0%)	7 (18.4%)	7 (19.4%)	0.942
Weight (g)	4278.84 (± 629.53)	4365.75 (± 835.52)	4449.97 (± 837.89)	0.706
Head Circumference (cm)	37.54 (± 1.22)	37.57 (± 1.39)	37.06 (± 5.18)	0.774
Height (cm)	54.06 (± 1.92)	54.57 (± 2.63)	53.63 (± 8.81)	0.776
BMI (kg/m^2^)	14.50 (± 1.37)	14.51 (± 1.81)	14.84 (± 1.52)	0.612
Fat-free mass (g)	3472.82 (± 419.75)	3538.21 (± 499.28)	3646.54 (± 419.62)	0.334
% Fat mass	17.94 (± 4.94)	18.96 (± 7.13)	18.24 (± 4.67)	0.776
Fat mass (g)	782.61 (± 286.27)	870.19 (± 423.85)	1096.31 (± 1570.20)	0.448
**2 months**	(*n* = 24)	(*n* = 39)	(*n* = 35)	*p*-value
Exclusive Breastfeeding	19 (79.2%)	31 (79.5%)	28 (80.0%)	0.997
Breastfeeding + formula	5 (20.8%)	6 (15.4%)	6 (17.1%)	0.857
Weight (g)	5510.38 (±632.56)	5464.03 (±876.13)	5578.17 (±798.23)	0.826
Head circumference (cm)	39.65 (± = 1.00)	39.37 (±1.43)	39.55 (±0.85)	0.628
Height (cm)	58.70 (±1.85)	58.34 (±2.41)	58.46 (±2.46)	0.837
BMI (kg/m^2^)	15.94 (±1.43)	15.96 (±1.88)	16.22 (±1.81)	0.764
Fat-free mass (g)	4144.91 (±415.53)	4158.05 (±516.82)	4248.54 (±427.62)	0.620
% Fat mass	23.88 (±5.93)	24.08 (±9.66)	23.26 (±4.78)	0.888
Fat mass (g)	1324.91(±415.69)	1307.64 (±480.49)	1329.86 (±445.69)	0.976
**4 months**	(*n* = 20)	(*n* = 31)	(*n* = 30)	*p*-value
Exclusive Breastfeeding	18 (80.0%)	20 (66.7%)	21 (70.0%)	0.583
Breastfeeding + formula	4 (20.0%)	8 (25.8%)	6 (20.0%)	0.830
Weight (g)	6808.70 (± 813.67)	6878.06 (± 776.91)	7019.33 (± 1017.07)	0.684
Head Circumference (cm)	42.01 (± 0.88)	41.61 (± 1.26)	41.90 (± 1.22)	0.453
Height (cm)	63.72 (± 2.07)	63.29 (± 2.22)	63.66 (± 2.24)	0.731
BMI (kg/m^2^)	16.74 (± 1.71)	17.15 (± 1.64)	17.27 (± 2.03)	0.591
Fat-free mass (g)	4820.75 (± 317.17)	4856.10 (± 668.01)	4943.32 (± 362.01)	0.715
% Fat mass	26.10 (± 5.07)	26.62 (± 4.69)	25.77 (± 3.95)	0.787
Fat mass (g)	1729.81 (± 471.62)	1954.21 (± 823.67)	1735.28 (± 377.22)	0.345

*BMI* Body mass index

The proportion of exclusively breastfed infants remained high in the follow-up study. In total, 79.8% of infants were exclusively breastfed at 1 month, 79.6% at 2 months and 71.3% at 4 months old.

Newborn FM at birth (grams) was higher in babies whose mothers were overweight before the pregnancy and who gained excessive gestational weight. However, no significant differences in % FM were observed.

The demographic data at birth of excluded newborn infants were similar to the data of those included. In addition, we were able to collect information about breastfeeding for most of the infants who were included but did not return for a follow-up at 4 months. Some of these infants returned after 4 months of age; however, we were not able to perform the plethysmography exam because they were not of the right length/weight suitable for the machine. Infants weighing more than 6 kg could not be evaluated with the PeaPod machine.

## Discussion

This study revealed that children of mothers with excessive weight gain during pregnancy had an increased percentage of body fat and birth weight. These results are similar to studies that related an adverse intrauterine environment to chronic noncommunicable diseases in adult life [6, 12, 21].

The prevalence of women with excessive gestational weight gain has been increasing worldwide [7]. Similar to the findings of this study, in which 41.1% of the women showed excessive gestational weight gain according to the 2009 IOM criteria [10], Goldstein et al. reported a prevalence of 47% [7], Hull et al. reported a prevalence of 53% [22], Starling et al. reported a prevalence of 51% [5], and Crozier et al. reported a prevalence of 49% [3].

It is assumed that in pregnant women with excessive weight gain, there is a positive energy balance with the accumulation of energy, increased insulin resistance and increased placental transport of glucose and fatty acids, which favors increased fetal growth, fetal fat deposition and adipocyte leptin synthesis [23]. The higher levels of leptin and fetal insulin can cause an alteration in the hypothalamus, leading to changes in appetite regulation [24]. Other previous studies have described that both this excessive weight gain and any other factor that modifies the intrauterine environment may trigger long-term effects in the offspring [10, 23, 25].

A systematic review and meta-analysis revealed that pregnant women with a weight gain exceeding the 2009 IOM [10] recommendations had an increased risk for newborns large for gestational age (OR 1.85), macrosomia (OR 1.95) and cesarean delivery (OR 1.30) [7]. In this study, although the newborn infants were heavier in the gestational overweight group than in the other groups, they were classified as adequate for gestational age. Additionally, the cesarean section rate was similar among the groups.

The evaluation of body composition in this study allowed for the early detection of FM, contributing to a better evaluation of growth after birth. Hull et al. also demonstrated and elevated FM in the newborns of mothers who gained excessive weight during pregnancy using a similar methodology [22]. Josefson et al. observed increased weight (*p*-value = 0.028), height (*p*-value = 0.026), FM (*p*-value = 0.009), % FM (*p*-value = 0.012), and serum leptin levels in the umbilical cord blood (*p*-value = 0.046) at birth in the group of women who gained excessive weight during pregnancy [26]. Other studies also observed a positive association between gestational weight gain and the newborn’s FM at birth [27] that persisted until 4 to 6 years of age [8]. Au et al. demonstrated through linear regression analysis that gestational weight gain was a variable that influenced neonatal adiposity [28].

In contrast, a study demonstrated that newborns from mothers with excessive gestational weight gain had an increased total FM and FFM but did not show an increased FM relative to the newborns from mothers with adequate gestational weight gain, suggesting that excessive gestational weight gain is more associated with total body mass than adiposity [5].

When we evaluated the body composition of newborns stratified by pregestational BMI of the mother, overweight women and those who gained excess weight during pregnancy had children with an increased FM in grams compared with mothers with adequate or insufficient weight gain (*p*-value = 0.037). These results were similar to those reported by Hull et al. [22].

The large proportion of exclusively breastfeed infants in this study could be justified by the fact that the Fernandes Figueira National Institute of Women, Children and Adolescent Health has a Human Milk Bank with a multidisciplinary team that supports breastfeeding after birth, in the well-baby nursery room, and in routine appointments after discharge.

The limitations of this study were that it did not assess the gestational period (first, second and third trimesters) with the highest maternal weight gain. A more significant weight gain in early gestation reflects increased maternal fat, whereas weight gain in the second and third trimesters of pregnancy is more related to fetal and placental growth and increased amniotic fluid volume [29]. It has been proposed that weight gain at the beginning of gestation, which initially represents an increase in maternal fat, may be associated with fetal adiposity due to the increased availability of glucose, amino acids, and maternal free fatty acids. However, further studies on neonatal body composition and specific periods of gestational weight gain are required [5].

Another limitation was the different loss of follow-up rates among the three weight gain groups after 4 months; there were no differences in growth or body composition at 1, 2 and 4 months of age, but it is not possible to exclude selection bias.

## Conclusions

The course of developing childhood obesity is influenced by a combination of pre- and postnatal factors. The changes in body composition at birth reported in this study suggest that the intrauterine environment is an influence.

Future studies with larger sample sizes and a more extended follow-up period are necessary to evaluate the influence of gestational weight gain on the long-term body composition of newborns as it relates to obesity and cardiovascular diseases.

## Data Availability

All the data and material are available by requesting to Research Department of IFF/Fiocruz.

## Abbreviations

BMI: Body mass index
FFM: Fat free mass
FM: Fat mass
IOM: Institute of medicine
OGTT: Oral Glucose tolerance test
OR: Odds ratio
TORCH: Toxoplasmosis, rubella, cytomegalovirus, herpes virus
WHO: World health organization
